# miRNA Repertoires of Demosponges *Stylissa carteri* and *Xestospongia testudinaria*

**DOI:** 10.1371/journal.pone.0149080

**Published:** 2016-02-12

**Authors:** Yi Jin Liew, Taewoo Ryu, Manuel Aranda, Timothy Ravasi

**Affiliations:** 1 Division of Biological and Environmental Sciences & Engineering, King Abdullah University of Science and Technology, Thuwal, 23955–6900, Kingdom of Saudi Arabia; 2 Red Sea Research Center, King Abdullah University of Science and Technology, Thuwal, 23955–6900, Kingdom of Saudi Arabia; 3 KAUST Environmental Epigenetics Program (KEEP), King Abdullah University of Science and Technology, Thuwal, 23955–6900, Kingdom of Saudi Arabia; University of New South Wales, AUSTRALIA

## Abstract

MicroRNAs (miRNAs) are small regulatory RNAs that are involved in many biological process in eukaryotes. They play a crucial role in modulating genetic expression of their targets, which makes them integral components of transcriptional regulatory networks. As sponges (phylum Porifera) are commonly considered the most basal metazoan, the in-depth capture of miRNAs from these organisms provides additional clues to the evolution of miRNA families in metazoans. Here, we identified the core proteins involved in the biogenesis of miRNAs, and obtained evidence for *bona fide* miRNA sequences for two marine sponges *Stylissa carteri* and *Xestospongia testudinaria* (11 and 19 respectively). Our analysis identified several miRNAs that are conserved amongst demosponges, and revealed that all of the novel miRNAs identified in these two species are specific to the class Demospongiae.

## Introduction

MicroRNAs (miRNAs) are small non-coding RNAs of about 22 nucleotides in length (Ha and Kim [1] details the biogenesis of these molecules). They play key roles in many physiological processes, such as development, cell proliferation and stress response, mainly through post-transcriptional degradation and translational repression of their mRNA targets [2, 3]. While studies have mainly focused on identifying novel miRNAs and uncovering their biological function in model metazoans, the ubiquity of next-generation sequencing and the completion of several invertebrate genomes have resulted in miRNAs being identified in more basal metazoans, e.g. in the sponge *Amphimedon queenslandica* [4] the cnidarians *Stylophora pistillata* [5], *Nematostella vectensis* [4, 6] and *Hydra magnipapillata* [7].

In this study, we identified the core RNAi proteins involved in the biogenesis of miRNAs in the draft proteome of the marine sponges *Stylissa carteri* and *Xestospongia testudinaria* from the Red Sea (Ryu et al., in review). The presence of a functional RNAi machinery lends more support to predicted miRNAs having important roles in both organisms. In order to identify *bona fide* miRNAs present in both sponges, we sequenced the small RNA fraction of both sponges and mapped the reads to the respective draft genomes (Ryu et al., in review). These miRNAs were subsequently compared to the previously described small RNAs set from *Amphimedon queenslandica* [4]. On top of identifying novel miRNAs for both *S*. *carteri* and *X*. *testudinaria*, by means of comparative analysis, we identified a subset of miRNAs that are conserved between the three studied poriferans. We believe our work would prove to be a valuable resource in understanding the role of miRNAs in the more basal branches of metazoans, and provide clues to why none of the poriferan miRNAs were conserved in other metazoans.

## Materials and Methods

### Ethics Statement

No special permissions were required for the collection of our sponges. Our research did not involve endangered or protected species.

### Identification of Core RNAi Proteins

The sequences of six protein families related to the small RNA pathways (Argonaute, Dicer, Drosha, HEN1, Pasha, and Piwi) were retrieved from the UniRef database [8] for five organisms (*Arabidopsis thaliana*, *Caenorhabditis elegans*, *Drosophila melanogaster*, *Homo sapiens*, and *Schizosaccharomyces pombe*). Sequences were further clustered using CD-HIT [9] at a threshold of 90% sequence identity with no length restrictions across species boundaries to remove fragmented protein sequences. The remaining representative sequences (46 in total) were queried against the predicted proteomes of *S*. *carteri* and *X*. *testudinaria* (Ryu et al., in review) to identify potential sponge homologues (BLASTP, e-values < 10^−10^). These homologues were then searched for protein domains using InterProScan v5 [10]. The domains deemed essential for the RNA machineries were as follows: the PAZ (PF02170) and Piwi (PF02171) domains for Argonaute and Piwi; a pair of RNase III domains (PF00636) for Dicer and Drosha; a double-stranded RNA binding domain (PF00035) for Pasha; and a methyltransferase domain (PF13847) for HEN1. Candidate homologues lacking these domains were discarded. Additional support for the identities of the candidate homologues was obtained by carrying out reciprocal BLASTP searches of the translated candidates against all proteins in the Swiss-Prot database [11] (S1 File).

For each key domain (PF02170, PF02171, PF00636, PF00035 and PF13847), Clustal Omega [12] was used to align domain sequences from candidate homologues against sequences from UniRef and other published studies (9 additional Argonaute, 6 Dicer, 4 Drosha, 5 HEN1, 5 Pasha, and 9 Piwi) [4, 5, 13, 14]. The alignments were visualised using Jalview [15]. Key residues were derived from the literature [16–21] (S1, S2, S3, S4, S5 and S6 Figs).

Phylogenies were constructed over entire sequences for each of the six protein families to provide additional support for the presence of the RNAi machinery in the sponges. For each protein family, candidate homologues from the sponges were aligned with selected metazoan sequences from [5] using MUSCLE [22]. Aligned regions of low quality were removed with TrimAl using the built-in “gappyout” parameter [23] (S2 File). ProtTest3 [24] was used to determine the best model for amino acid substitutions, while MEGA v6 [25] was used to construct maximum-likelihood phylogenetic trees (applying the substitution model of “LG+G” for Argonaute, Drosha, and HEN1; and “LG+I+G” for Piwi, Dicer, and Pasha). Support values were computed from 1,000 bootstrap replicates.

### Searches for Other RNAi Proteins

We broadened our search to include four other RNAi proteins previously implicated in miRNA biogenesis in some metazoans: HYL1, Serrate, GW182, and RdRP (RNA-dependent RNA polymerase). Sequences for HYL1, Serrate and GW182 (13 in total) from four cnidarians (*Acropora digitifera*, *Acropora millepora*, *Hydra vulgaris*, and *Nematostella vectensis*) were obtained from Moran et al. [13]. For RdRP sequences (7 in total), they were retrieved from UniRef for the same five organisms detailed previously, analogous to how core RNAi protein sequences were obtained. These sequences were subsequently searched against the proteomes of *S*. *carteri* and *X*. *testudinaria*. As these RNAi proteins play less crucial roles, we did not impose the same strict requirements for the identification of homologues (e.g. presence of key domains, conservation of crucial amino acid residues).

Similarly, phylogenies were constructed by aligning and trimming low-quality sequences for Serrate and GW182 proteins (S3 File). The “JTT+G” and “LG+G” models respectively were used to construct the trees.

### Small RNA Library Construction

*S*. *carteri* and *X*. *testudinaria* specimens were collected on 18 September 2011 and 28 March 2012 via SCUBA at Fsar Reef (22.228408N, 39.028187E) at a depth of 13–14 m on the Red Sea coast of Saudi Arabia (Table 1). The specimens were washed three times in calcium-magnesium-free artificial seawater (CMF-ASW) and subsequently flash frozen in dry ice. Small RNAs were extracted with the mirVana miRNA Isolation Kit (Ambion, Austin, TX). RNA quality was assessed using a Bioanalyzer 2100 (Agilent Technologies, Santa Clara, CA). Small RNA libraries were prepared with TruSeq Small RNA protocol (Illumina, San Diego, CA) on targeted size ranges (18–40 and 18–70 bp). Low-quality ends (Phred score < 20), sequencing adapters, and short reads (< 16 bp) were removed using custom Java scripts.

**Table 1:** The statistics of reads used for the miRNA identification.

Species	Sampling date	Number of reads	Size of dataset (bp)
*S*. *carteri*	18 Sep 2011	4,754,232	152,135,424
	18 Sep 2011	3,175,330	101,610,560
	18 Sep 2011	4,968,941	159,006,112
	18 Sep 2011	3,972,657	127,125,024
	18 Sep 2011	5,286,305	169,161,760
	18 Sep 2011	4,147,847	132,731,104
	28 Mar 2012	15,793,662	1,105,556,340
	28 Mar 2012	10,438,070	730,664,900
	28 Mar 2012	18,735,440	1,311,480,800
*X*. *testudinaria*	18 Sep 2011	7,073,036	226,337,152
	18 Sep 2011	5,524,159	176,773,088
	18 Sep 2011	6,808,087	217,858,784
	18 Sep 2011	5,147,411	164,717,152
	18 Sep 2011	6,469,476	207,023,232
	18 Sep 2011	5,091,833	162,938,656
	28 Mar 2012	6,020,023	421,401,610
	28 Mar 2012	3,900,527	273,036,890
	28 Mar 2012	3,973,334	278,133,380

### Processing of Small RNA Reads

Due to the significant sequencing depth of the small RNA libraries obtained from both sponges (52.1 and 36.2 million reads for *S*. *carteri* and *X*. *testudinaria*, respectively), several filters were applied to remove sequences that might result from sequencing errors. First, reads that occurred less than five times in each library was removed. This step removed 8.8 million reads (17.0%) for *S*. *carteri*, and 11.5 million reads (31.7%) for *X*. *testudinaria*. A BLASTN search of the remaining reads was performed against Rfam v11 [26], a database of known noncoding RNA families. Reads that matched (e-value < 10^−5^) a known noncoding RNA family—most commonly to tRNAs and rRNAs—were excluded from further analyses. The removed reads included 49 “miRNA-like” reads (9 similar to let-7, and 40 similar to miR-10); however, none mapped to a genomic sequence that folded into a hairpin structure, indicating that they could be disregarded. This step removed a further 6.2 million reads (11.8%) from *S*. *carteri* and 3.2 million reads (9.7%) from *X*. *testudinaria*.

### miRNA Prediction and Filtering

miRDeep2 [27] was used to identify miRNA genes by mapping the small RNA reads for each sponge to their respective genomes using Bowtie v1.0.1 [28]. Potential pre-miRNA precursor sequences were identified by their mapping patterns and validated using RNAfold to confirm that they have the canonical hairpin loop structure [27]. Each predicted pre-miRNA that had a miRDeep2 score > 5 was retained for further analysis and inspected manually. A Python script was written to produce additional information not found in the miRDeep2 output (i.e., the length of the 3’ overhang, and the proportion of reads with a consistent 5’ end). Based on the output, we further selected a set of *bona fide* miRNAs. Conserved miRNAs were identified using BLASTN against all previously identified pre-miRNA sequences in miRBase v20 [29].

## Results

### Identification of Core RNAi Proteins

Six key proteins that are essential for miRNA processing and function were identified in both *S*. *carteri* and *X*. *testudinaria*–both sponges had homologues corresponding to one Argonaute, one Piwi, two Dicers, and one Drosha; *S*. *carteri* had one additional Pasha homologue, while *X*. *testudinaria* had one additional HEN1 homologue. These homologues were identified via an initial BLASTP search against known RNAi proteins, followed by the checking of protein domains crucial for catalytic activity, and lastly a reciprocal BLAST search against manually curated proteins in Swiss-Prot (S1 File).

**Fig 1:**
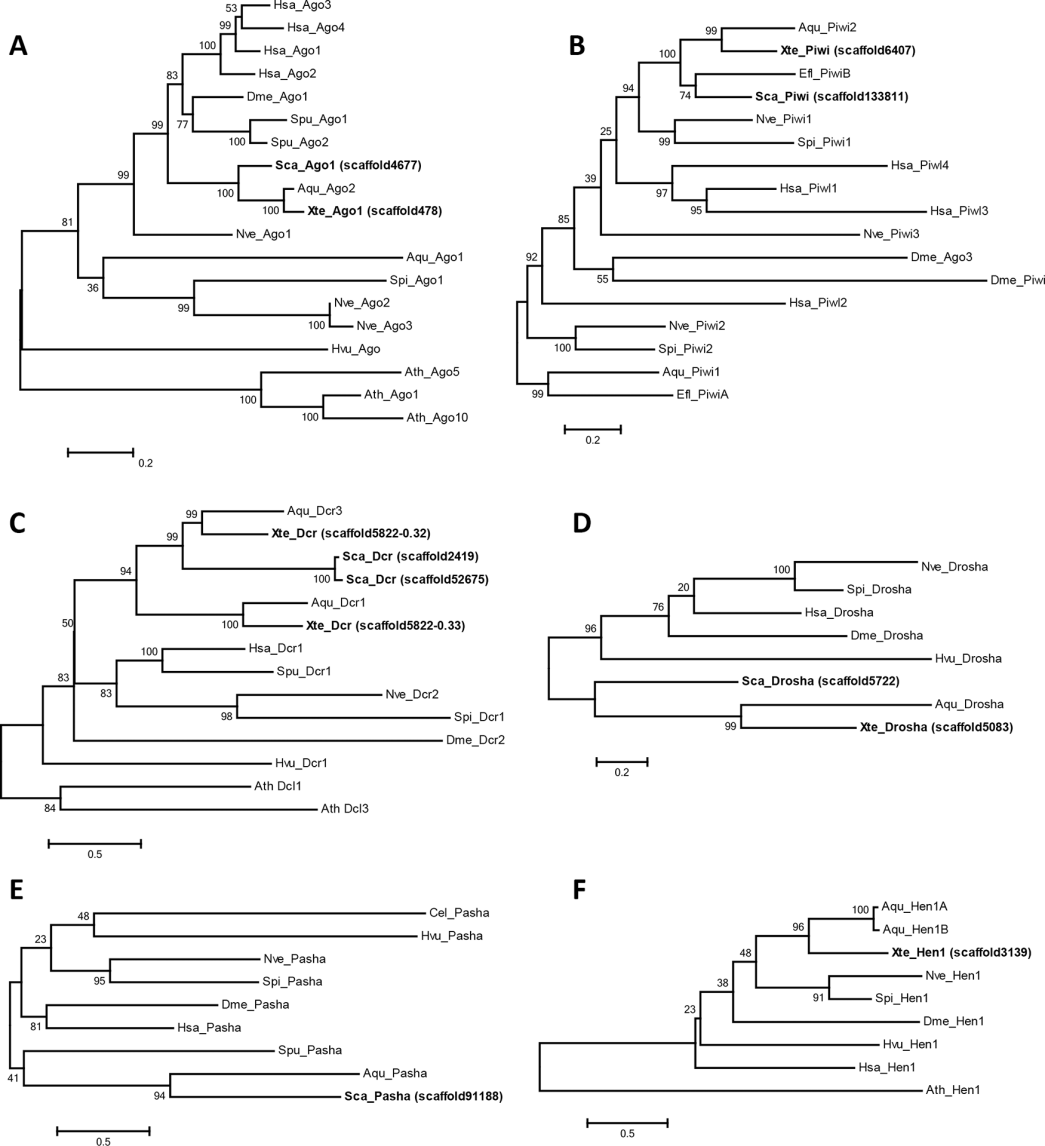
Maximum-likelihood phylogenies of the core proteins involved in small RNA biogenesis. Trees were constructed for (A) Argonaute, (B) Piwi, (C) Dicer, (D) Drosha, (E) Pasha, and (F) HEN1. (A), (D), and (F) were constructed using the LG+G amino acid substitution model, while (B), (C), and (E) were constructed using the LG+I+G model. Bootstrap support values are indicated above the branches. Species abbreviations: Ath, *Arabidopsis thaliana*; Aqu, *Amphimedon queenslandica*; Cel, *Caenorhabditis elegans*; Dme, *Drosophila melanogaster*; Efl, *Ephydatia fluviatilis*; Hsa, *Homo sapiens*; Hvu, *Hydra vulgaris*; Nve, *Nematostella vectensis*; Sca, *S*. *carteri*; Spi, *Stylophora pistillata*; Spu, *Strongylocentrotus purpuratus*; and Xte, *X*. *testudinaria*. *A*. *thaliana* sequences were selected as the outgroup when available. For Piwi, Drosha, and Pasha (whose protein families are not found in plants), sponge sequences were chosen as the outgroup, as they phylogenetically form the most basal clade in the tree.

The per-family alignments of candidate homologues against the known RNAi proteins showed that many of the functionally important amino acid residues within the key protein domains were conserved in the candidate homologues obtained from the two sponges. Examples include the strong conservation of charged amino acids (arginine, lysine, and glutamate) in the PAZ domain and the DDX triad in the Piwi domain of the Argonaute and Piwi homologues, as well as the aspartate and glutamate residues in the Dicer and Drosha homologues (S1, S2, S3, S4, S5 and S6 Figs). Maximum-likelihood phylogenetic trees for all six protein families (Fig 1A–1F) placed all of the candidate *S*. *carteri* and *X*. *testudinaria* homologues with those from either *A*. *queenslandica* or *Ephydatia fluviatilis*, strongly indicating that the RNAi machineries identified in both sponges are *bona fide*.

In addition to the core small RNA pathway proteins, we searched for homologues of HYL1, Serrate, GW182, and RdRP in both sponges. HYL1 and Serrate are known to be involved in converting pri-miRNAs to pre-miRNAs in plants [30], but full-length homologues of both proteins have also been reported in four cnidarians (*A*. *digitifera*, *A*. *millepora*, *H*. *vulgaris*, and *N*. *vectensis*) [13]. Homologues of GW182, which help Argonaute repress its targets [31], have also been identified in these four cnidarians. RdRPs use small RNAs as templates and contribute to amplifying their silencing effects by directing the production of secondary dsRNAs [32]. To date, functional RdRPs have been identified in plants [33, 34], *C*. *elegans* [35], and *N*. *vectensis* [36], but not in mammals nor flies. In the present work, we identified three Serrate homologues in *S*. *carteri*, one Serrate homologue and two GW182 homologues in *X*. *testudinaria*, and one Serrate homologue and one GW182 homologue in *A*. *queenslandica*. With the exception of one GW182 homologue in *X*. *tetsudinaria*, all of these homologues clustered best with other sequences of sponge origin (S7 Fig). We failed to find any homologues for HYL1 or RdRP in the three sponges, nor did we find homologues of GW182 in *S*. *carteri* (BLASTP e-value < 10^−10^; data not shown). The absence of HYL1 and RdRPs in sponges hints at the selective loss of these two protein families after the divergence of the phyla Porifera and Cnidaria.

### Identification of *Bona Fide* miRNAs

Roughly half of the filtered reads were successfully mapped to the corresponding genome (20.5 million reads out of 43.3 million for *S*. *carteri*; and 13.0 million reads out of 24.7 million reads for *X*. *testudinaria*). Based on these reads, 13 miRNA genes were predicted for *S*. *carteri*, 11 of which passed our additional filtering criteria; and 46 miRNA genes were predicted for *X*. *testudinaria*, with 19 passing the additional criteria (Table 2; S1 and S2 Tables). Out of the combined 30 miRNA predictions, 8 from *S*. *carteri* and 5 from *X*. *testudinaria* were found to match miRNAs that had been previously identified in other demosponges (*A*. *queenslandica* [4]; *Haliclona* sp. [6]; and *Dysidea camera*, *Clathria prolifera*, and *Suberites* sp. [37]). Like the other demosponges, all of the predicted miRNAs in *S*. *carteri* and *X*. *testudinaria* were class-specific; there was also no overlap between our predictions and the recently-discovered miRNAs in Calcisponges [38].

**Table 2:** List of 11 *bona fide* miRNAs in *S*. *carteri* and 19 in *X*. *testudinaria*. Abbreviations used are ‘aqu’: *A*. *queenslandica*; ‘sca’: *S*. *carteri*; ‘xte’: *X*. *testudinaria*.

miRNA name^1^	Predicted mature miRNA (5’– 3’)	Matches to known miRNAs
sca-mir-temp-1	uagauugggcuuggucggcaga	aqu-miR-2016
sca-mir-temp-2	uagauugggcuuggucggcagg	aqu-miR-2016
sca-mir-temp-3	ugguggucgguguuuugugga	aqu-miR-2021
sca-mir-temp-4	aaagugaucggguugccgucu	aqu-miR-2019
sca-mir-temp-5	ucauguauuguggaggggaga	aqu-miR-2015
sca-mir-temp-6	uaccugugcaccugugugccc	aqu-miR-2017
sca-mir-temp-7	uggguagugugucuuuucgga	aqu-miR-2020
sca-mir-temp-8	cggggcguggcgcagcc	
sca-mir-temp-11	aauagaaccacgauacagcuugccu	
sca-mir-temp-12	cucucaagcucugaauaagcu	
sca-mir-temp-13	ugucggagccggagguuc	aqu-miR-2018
xte-mir-temp-1	uagauugggcuuggucggcaga	aqu-miR-2016
xte-mir-temp-2	aaagugaucggguugccgucu	aqu-miR-2019
xte-mir-temp-3	cagauggacagaguagagagu	
xte-mir-temp-4	ucauguauuguggaggggaga	aqu-miR-2015
xte-mir-temp-5	auauagauaccaauaggauu	
xte-mir-temp-6	ugguggucgguguuucgugga	aqu-miR-2021
xte-mir-temp-7	gagauggacacaguaaagagu	
xte-mir-temp-8	uggguagugugucuuuucgga	aqu-miR-2020
xte-mir-temp-12	ccggccaugccuucguacc	
xte-mir-temp-16	accccucugcugcuuuuuug	
xte-mir-temp-19	caauucuggauuccggc	
xte-mir-temp-20	uuccaaucccgauuacguagu	
xte-mir-temp-27	auccuguucauccauguaa	
xte-mir-temp-31	auccuguucauccauguaa	
xte-mir-temp-34	acagcaucuggguggacacc	
xte-mir-temp-35	ugccccguacauagccucugauu	
xte-mir-temp-39	agggacgcagagcgcuc	
xte-mir-temp-44	cucuaccgcugaugaggaggcacu	
xte-mir-temp-46	ugaacugugauuccugggau	

^1^ Note to reviewers: the nomenclature of these miRNAs are temporary. miRBase (the miRNA registry) only accepts submissions of new miRNAs after the manuscript is accepted for publication.

**Fig 2:**
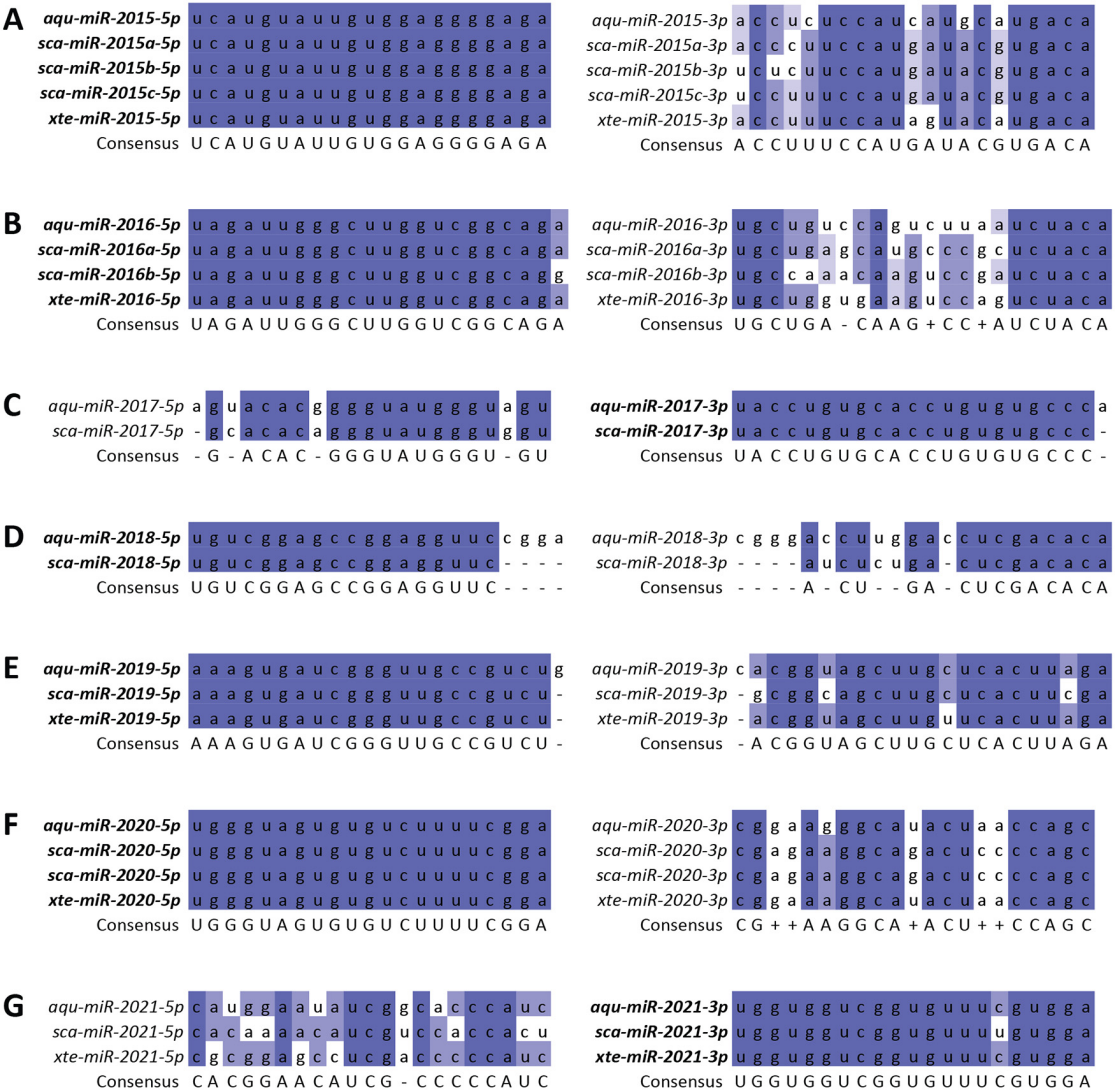
Alignments of predicted *S*. *carteri* and *X*. *testudinaria* miRNAs against known *A*. *queenslandica* miRNAs. Homologues of (A) aqu-miR-2015, (B) aqu-miR-2016, (C) aqu-miR-2017, (D) aqu-miR-2018, (E) aqu-miR-2019, (F) aqu-miR-2020, and (G) aqu-miR-2021 were identified in *S*. *carteri* and *X*. *testudinaria*. The miRNA sequences from the 5’ and 3’ arms are presented on the left and right, respectively. The bolded name denotes the arm that had more reads mapped to it (i.e., the mature arm for that miRNA). Bases are coloured to enable visualisation of the conservation level, as follows: dark blue, > 80%; blue, > 60%; light blue, > 40%; uncoloured, ≤ 40%). The abbreviations are as described in Fig 1.

Comparisons between the three demosponges with complete pre-miRNA sequences (i.e. both the mature and the star sequences) reveal the strong conservation of miRNA repertoires across the three sponges: seven of the eight miRNAs identified in *A*. *queenslandica* had homologues in either *S*. *carteri* or *X*. *testudinaria* (Fig 2). Of the seven, five (miR-2015, miR-2016, miR-2019, miR-2020, miR-2021) were present in both sponges; the other two (miR-2017, miR-2018) were present only in *S*. *carteri* but not in *X*. *testudinaria*. For these homologues, the mature sequences are almost always fully conserved, with at most a one-base mismatch between species; the star sequences have, as expected, more mismatches, which is in line with the current understanding that star miRNAs are less functionally constrained.

It is interesting to note that the co-expression pattern of the mature reads from the two arms of aqu-miR-2015 (~2,700 reads mapped to each arm of the miRNA hairpin [4]) differed between *S*. *carteri* and *X*. *testudinaria*. For *S*. *carteri*, the 5’ and 3’ arms were mapped by 34,026 and 2,283 reads, respectively, while for *X*. *testudinaria*, the 5’ and 3’ arms were mapped by 45,845 and 447 reads, respectively. This observation is corroborated by the complete conservation of the 5’ arm across all three sponges, while there were several mismatches in the 3’ arm. (Fig 2). This suggests that miR-2015-5p has similar biological function across all three sponges, while miR-2015-3p is likely to be only functional in *A*. *queenslandica*, but not in the other two sponges. For all of the other conserved miRNAs in *S*. *carteri* and *X*. *testudinaria*, the relative abundances of the miRNAs derived from both arms of the hairpin loop were similar to those observed in *A*. *queenslandica*.

## Discussion

### Identification of Core RNAi Proteins

Previously, Grimson et al. [4] had identified the presence of a functional small RNA machinery in *A*. *queenslandica*. In this study, we identified candidate homologues of core RNAi proteins in the *S*. *carteri* and *X*. *testudinaria* proteomes, strongly indicating that small RNA regulation is universal even in the most basal metazoans. These homologues, when aligned against known RNAi proteins, revealed fairly strong conservation of key protein domains and residues crucial for protein function; also, maximum-likelihood phylogenetic trees of our candidate homologues with other known RNAi proteins provided additional support that the identified homologues are poriferan in nature. As expected, all of these candidate homologues clustered strongly with sequences from *A*. *queenslandica* or *E*. *fluviatilis*, producing branch scores that are close to 100.

### miRNA Repertoires of *S*. *carteri* and *X*. *testudinaria*

We predicted a combined set of 30 *bona fide* miRNAs for both sponges, including seven of the eight miRNAs previously identified in other demosponges [4, 6, 37]. Five of these conserved miRNAs were present in both *S*. *carteri* and *X*. *testudinaria*, while the other two is present in *S*. *carteri*. Consistent with previous observations by other groups [4, 6, 37–39], all of our predicted miRNAs were specific to the class Demospongiae. Also, the novel miRNAs in *S*. *carteri* and *X*. *testudinaria* were unique to those species, indicating that miRNA genes continued to expand after the evolutionary divergence of both species.

Based on the phylogenetic tree in Sperling et al. [37], as well as Northern blots in Wheeler et al. [6], there is good reason to believe that all eight miRNAs originally identified in *A*. *queenslandica* (i.e. miR-2014 to miR-2021) should also be present in *S*. *carteri* and *X*. *testudinaria*, as *S*. *carteri* is a democlavid while *X*. *testudinaria* is a haplosclerid. Consistent with this expectation, with the exception of miR-2021 in *S*. *carteri*, we found matching reads in *S*. *carteri* and *X*. *testudinaria* to all eight aforementioned miRNAs (Table 3). However, as our miRNA prediction pipeline is dependent on the short reads in the library mapping in a hairpin-like manner against the draft genome, it is possible that the current limitations of the genomes we used contributed to the inability to predict more conserved miRNAs from both of our short read datasets. This was more evident for *X*. *testudinaria*: the mature and star sequences of miR-2014 maps perfectly in close proximity to the same *X*. *testudinaria* scaffold, but unfortunately, these loci were separated by a stretch of Ns; miR-2017-3p and miR-2018-5p did not have any matches to the genome. Additional PCR verification with miRNA sequences as primers succeeded in amplifying miR-2014 (S8 Fig); on the other hand, miR-2017 and miR-2018 did not amplify, in line with their absence in the genome. For *S*. *carteri*, the mature miR-2014 identified in *A*. *queenslandica* (aqu-miR-2014-3p) corresponds perfectly to the star sequence of sca-miR-temp-2 –the mature sequence of sca-miR-temp-2 is, intriguingly, a homologue of aqu-miR-2016-5p (S1 Table).

**Table 3:** Number of reads present in *S*. *carteri* and *X*. *testudinaria* that matches known sequences in *A*. *queenslandica*. miRNAs in bold are the mature miRNA, while the unbolded ones are star sequences.

	Exact matches in…	
*A*. *queenslandica* miRNA	*S*. *carteri*	*X*. *testudinaria*
aqu-miR-2014-5p	-	6,097
**aqu-miR-2014-3p**	**4,799**	**11,741**
**aqu-miR-2015-5p**	**18,603**	**21,111**
aqu-miR-2015-3p	-	-
**aqu-miR-2016-5p**	**96,701**	**964,750**
aqu-miR-2016-3p	-	-
aqu-miR-2017-5p	-	-
**aqu-miR-2017-3p**	**1,288**	**4,398**
**aqu-miR-2018-5p**	**385**	**9,379**
aqu-miR-2018-3p	-	-
**aqu-miR-2019-5p**	**6,453**	**8,937**
aqu-miR-2019-3p	-	-
**aqu-miR-2020-5p**	**1,127**	**4,165**
aqu-miR-2020-3p	-	-
aqu-miR-2021-5p	-	-
**aqu-miR-2021-3p**	**-**	**11,937**

Generally speaking, our data is in line with the consensus that the larger eumetazoan miRNA repertoire—relative to sponges—contributes to the increased morphological complexity seen in eumetazoans [6, 39–41]. However, the absence of miR-2021 in *S*. *carteri* and the presence of new miRNAs in both *S*. *carteri* and *X*. *testudinaria* can be construed as the evidence that miRNAs have been gained and lost in sponges despite their relatively simple body structures and lifestyles.

In conclusion, on top of demonstrating the strong conservation of a subset of miRNAs across demosponges, we have also expanded the known set of high confidence demosponge miRNAs into the double digits. We provide further indication that miRNA expansions and losses continued even after the evolutionary divergence of the orders within demosponges.

## Data Accessibility

The sequencing datasets for *S*. *carteri* and *X*. *testudinaria* are available under NCBI BioProject IDs of PRJNA254402 and PRJNA254412, respectively.

## Supporting Information

S1 Fig Graphical alignment of the PAZ domains in Argonaute and Piwi proteins. Of note are the strong conservation of phenylalanine (F) at position 43 and glutamate (E) at position 113. Mutations in the former affect RNA binding; mutations in the latter produce insoluble protein. Key residue positions are marked with red asterisks.
(TIF)

S2 Fig Graphical alignment of the Piwi domains in Argonaute and Piwi proteins. The catalytic DDX triad, which contributes to the slicing activity of the ribonuclease (marked in red asterisks), is located at positions 51, 140 and 284 or positions 51, 140 and 154. This triad is absent in the candidate Piwi homologue for *X*. *testudinaria*, likely due to it being truncated.
(TIF)

S3 Fig Graphical alignment of the first RNase III domain in Dicer and Drosha proteins. The acidic aspartate (D) and glutamate (E) residues are involved in the coordination of a divalent metal cation, and are well conserved in most of the candidate homologues. These residues are absent in the candidate Dicers for *S*. *carteri*, likely due to truncation.
(TIF)

S4 Fig Graphical alignment of the second RNase III domain in Dicer and Drosha proteins. The aspartate (D) and glutamate (E) residues involved in the coordination of a divalent metal cation are perfectly conserved across all candidate Dicers and Drosha proteins.
(TIF)

S5 Fig Graphical alignment of the dsRNA-binding domain in Pasha. The conventional alanine/alanine pair (AA, positions 21 and 22) and alanine/serine pair (AS, positions 161 and 162) involved in the binding of dsRNA are not present in the candidate Pasha for *S*. *carteri*. Instead, for the former, *S*. *carteri* Pasha has an isoleucine/alanine pair that is not found in any other known Pasha; for the latter, it has a glycine/alanine pair, which is an exact match of *S*. *purpuratus* Pasha.
(TIF)

S6 Fig Graphical alignment of the methyltransferase domain in HEN1. The residues involved in Mg^2+^ coordination (positions 113, 116, 117, and 118) are well-conserved across the aligned sequences; residues associated with the cofactor AdoHcy and 3' terminus (other positions marked by a red asterisk) are absent, likely due to the candidate being truncated.
(TIF)

S7 Fig Maximum-likelihood phylogenies of Serrate and GW182 proteins in four cnidarians and three sponges. The (A) Serrate tree was built with the LG+G amino acid substitution model, while (B) GW182 was with the JTT+G model.
(TIF)

S8 Fig PCR-based confirmation of xte-miR-2014. Primers corresponding to miR-2014-5p and the reverse complement of miR-2014-3p successfully amplified a ~100bp fragment in two *X*. *testudinaria* samples. Sanger sequences from the bands, when aligned against aqu-mir-2014 from *A*. *queenslandica* and the genomic sequence from *X*. *testudinaria* containing xte-mir-2014, indicate that the stretch of Ns in the *X*. *testudinaria* genome is an artefact from genome assembly, possibly hindering the *in silico* identification of this miRNA.
(TIF)

S1 File Candidate RNAi proteins in *S*. *carteri* and *X*. *testudinaria*. (DOCX)

S2 File Sequences (in FASTA) that were used to construct the maximum-likelihood phylogenetic trees for core RNAi proteins. (FA)

S3 File Sequences (in FASTA) that were used to construct the maximum-likelihood phylogenetic trees for Serrate and GW182. (FA)

S1 Table Additional criteria used to filter for *bona fide* miRNAs in *S*. *carteri* from default miRDeep2 output. (XLSX)

S2 Table Additional criteria used to filter for *bona fide* miRNAs in *X*. *testudinaria* from default miRDeep2 output. (XLSX)

## References

[1] Ha M, Kim VN. Regulation of microRNA biogenesis. Nature reviews Molecular cell biology. 2014;15(8):509–24. Epub 2014/07/17. 10.1038/nrm3838 . 25027649

[2] Nehammer C, Podolska A, Mackowiak SD, Kagias K, Pocock R. Specific microRNAs regulate heat stress responses in Caenorhabditis elegans. Scientific reports. 2015;5:8866 Epub 2015/03/10. 10.1038/srep08866 ; PubMed Central PMCID: PMCPmc4352874. 25746291 PMC4352874

[3] Vidigal JA, Ventura A. The biological functions of miRNAs: lessons from in vivo studies. Trends in cell biology. 2015;25(3):137–47. Epub 2014/12/09. 10.1016/j.tcb.2014.11.004 ; PubMed Central PMCID: PMCPmc4344861. 25484347 PMC4344861

[4] Grimson A, Srivastava M, Fahey B, Woodcroft BJ, Chiang HR, King N, et al Early origins and evolution of microRNAs and Piwi-interacting RNAs in animals. Nature. 2008;455(7217):1193–7. Epub 2008/10/03. 10.1038/nature07415 . 18830242 PMC3837422

[5] Liew YJ, Aranda M, Carr A, Baumgarten S, Zoccola D, Tambutte S, et al Identification of MicroRNAs in the Coral Stylophora pistillata. PLoS One. 2014;9(3):e91101 Epub 2014/03/25. 10.1371/journal.pone.0091101 ; PubMed Central PMCID: PMCPmc3962355. 24658574 PMC3962355

[6] Wheeler BM, Heimberg AM, Moy VN, Sperling EA, Holstein TW, Heber S, et al The deep evolution of metazoan microRNAs. Evol Dev. 2009;11(1):50–68. Epub 2009/02/07. 10.1111/j.1525-142X.2008.00302.x . 19196333

[7] Chapman JA, Kirkness EF, Simakov O, Hampson SE, Mitros T, Weinmaier T, et al The dynamic genome of Hydra. Nature. 2010;464(7288):592–6. Epub 2010/03/17. 10.1038/nature08830 ; PubMed Central PMCID: PMCPmc4479502. 20228792 PMC4479502

[8] Suzek BE, Huang H, McGarvey P, Mazumder R, Wu CH. UniRef: comprehensive and non-redundant UniProt reference clusters. Bioinformatics. 2007;23(10):1282–8. Epub 2007/03/24. 10.1093/bioinformatics/btm098 . 17379688

[9] Li W, Godzik A. Cd-hit: a fast program for clustering and comparing large sets of protein or nucleotide sequences. Bioinformatics. 2006;22(13):1658–9. Epub 2006/05/30. 10.1093/bioinformatics/btl158 . 16731699

[10] Mulder N, Apweiler R. InterPro and InterProScan: tools for protein sequence classification and comparison. Methods in molecular biology (Clifton, NJ). 2007;396:59–70. 10.1007/978-1-59745-515-2_5 18025686

[11] Magrane M, Consortium U. UniProt Knowledgebase: a hub of integrated protein data. Database (Oxford). 2011;2011:bar009 Epub 2011/03/31. 10.1093/database/bar009 ; PubMed Central PMCID: PMCPmc3070428. 21447597 PMC3070428

[12] Sievers F, Wilm A, Dineen D, Gibson TJ, Karplus K, Li W, et al Fast, scalable generation of high-quality protein multiple sequence alignments using Clustal Omega. Mol Syst Biol. 2011;7:539 Epub 2011/10/13. 10.1038/msb.2011.75 ; PubMed Central PMCID: PMCPmc3261699. 21988835 PMC3261699

[13] Moran Y, Praher D, Fredman D, Technau U. The Evolution of MicroRNA Pathway Protein Components in Cnidaria. Mol Biol Evol. 2013 Epub 2013/09/14. 10.1093/molbev/mst159 . 24030553 PMC3840309

[14] Rodriguez AJ, Seipel SA, Hamill DR, Romancino DP, M DIC, Suprenant KA, et al Seawi—a sea urchin piwi/argonaute family member is a component of MT-RNP complexes. Rna. 2005;11(5):646–56. Epub 2005/04/21. 10.1261/rna.7198205 ; PubMed Central PMCID: PMCPmc1370751. 15840816 PMC1370751

[15] Waterhouse AM, Procter JB, Martin DM, Clamp M, Barton GJ. Jalview Version 2—a multiple sequence alignment editor and analysis workbench. Bioinformatics. 2009;25(9):1189–91. Epub 2009/01/20. 10.1093/bioinformatics/btp033 ; PubMed Central PMCID: PMCPmc2672624. 19151095 PMC2672624

[16] Huang Y, Ji L, Huang Q, Vassylyev DG, Chen X, Ma JB. Structural insights into mechanisms of the small RNA methyltransferase HEN1. Nature. 2009;461(7265):823–7. Epub 2009/10/09. 10.1038/nature08433 . 19812675 PMC5125239

[17] Lee YS, Nakahara K, Pham JW, Kim K, He Z, Sontheimer EJ, et al Distinct roles for Drosophila Dicer-1 and Dicer-2 in the siRNA/miRNA silencing pathways. Cell. 2004;117(1):69–81. Epub 2004/04/07. . 15066283 10.1016/s0092-8674(04)00261-2

[18] Lingel A, Simon B, Izaurralde E, Sattler M. Structure and nucleic-acid binding of the Drosophila Argonaute 2 PAZ domain. Nature. 2003;426(6965):465–9. Epub 2003/11/15. 10.1038/nature02123 . 14615801

[19] Rivas FV, Tolia NH, Song JJ, Aragon JP, Liu J, Hannon GJ, et al Purified Argonaute2 and an siRNA form recombinant human RISC. Nat Struct Mol Biol. 2005;12(4):340–9. Epub 2005/04/01. 10.1038/nsmb918 . 15800637

[20] Song JJ, Smith SK, Hannon GJ, Joshua-Tor L. Crystal structure of Argonaute and its implications for RISC slicer activity. Science. 2004;305(5689):1434–7. Epub 2004/07/31. 10.1126/science.1102514 . 15284453

[21] Yeom KH, Lee Y, Han J, Suh MR, Kim VN. Characterization of DGCR8/Pasha, the essential cofactor for Drosha in primary miRNA processing. Nucleic Acids Res. 2006;34(16):4622–9. Epub 2006/09/12. 10.1093/nar/gkl458 ; PubMed Central PMCID: PMCPmc1636349. 16963499 PMC1636349

[22] Edgar RC. MUSCLE: multiple sequence alignment with high accuracy and high throughput. Nucleic Acids Res. 2004;32(5):1792–7. Epub 2004/03/23. 10.1093/nar/gkh340 ; PubMed Central PMCID: PMCPmc390337. 15034147 PMC390337

[23] Capella-Gutierrez S, Silla-Martinez JM, Gabaldon T. trimAl: a tool for automated alignment trimming in large-scale phylogenetic analyses. Bioinformatics. 2009;25(15):1972–3. Epub 2009/06/10. 10.1093/bioinformatics/btp348 ; PubMed Central PMCID: PMCPmc2712344. 19505945 PMC2712344

[24] Darriba D, Taboada GL, Doallo R, Posada D. ProtTest 3: fast selection of best-fit models of protein evolution. Bioinformatics. 2011;27(8):1164–5. Epub 2011/02/22. 10.1093/bioinformatics/btr088 . 21335321 PMC5215816

[25] Tamura K, Stecher G, Peterson D, Filipski A, Kumar S. MEGA6: Molecular Evolutionary Genetics Analysis version 6.0. Mol Biol Evol. 2013;30(12):2725–9. Epub 2013/10/18. 10.1093/molbev/mst197 ; PubMed Central PMCID: PMCPmc3840312. 24132122 PMC3840312

[26] Burge SW, Daub J, Eberhardt R, Tate J, Barquist L, Nawrocki EP, et al Rfam 11.0: 10 years of RNA families. Nucleic Acids Res. 2013;41(Database issue):D226–32. Epub 2012/11/06. 10.1093/nar/gks1005 ; PubMed Central PMCID: PMCPmc3531072. 23125362 PMC3531072

[27] Friedlander MR, Mackowiak SD, Li N, Chen W, Rajewsky N. miRDeep2 accurately identifies known and hundreds of novel microRNA genes in seven animal clades. Nucleic Acids Res. 2012;40(1):37–52. Epub 2011/09/14. 10.1093/nar/gkr688 ; PubMed Central PMCID: PMCPmc3245920. 21911355 PMC3245920

[28] Langmead B, Trapnell C, Pop M, Salzberg SL. Ultrafast and memory-efficient alignment of short DNA sequences to the human genome. Genome biology. 2009;10(3):R25–R. 10.1186/gb-2009-10-3-r25 19261174 PMC2690996

[29] Kozomara A, Griffiths-Jones S. miRBase: integrating microRNA annotation and deep-sequencing data. Nucleic Acids Res. 2011;39(Database issue):D152–7. Epub 2010/11/03. 10.1093/nar/gkq1027 ; PubMed Central PMCID: PMCPmc3013655. 21037258 PMC3013655

[30] Voinnet O. Origin, biogenesis, and activity of plant microRNAs. Cell. 2009;136(4):669–87. Epub 2009/02/26. 10.1016/j.cell.2009.01.046 . 19239888

[31] Huntzinger E, Izaurralde E. Gene silencing by microRNAs: contributions of translational repression and mRNA decay. Nat Rev Genet. 2011;12(2):99–110. Epub 2011/01/20. 10.1038/nrg2936 . 21245828

[32] Ghildiyal M, Zamore PD. Small silencing RNAs: an expanding universe. Nat Rev Genet. 2009;10(2):94–108. Epub 2009/01/17. 10.1038/nrg2504 ; PubMed Central PMCID: PMCPmc2724769. 19148191 PMC2724769

[33] Chen X. Small RNAs and their roles in plant development. Annu Rev Cell Dev Biol. 2009;25:21–44. Epub 2009/07/07. 10.1146/annurev.cellbio.042308.113417 . 19575669 PMC5135726

[34] Song X, Wang D, Ma L, Chen Z, Li P, Cui X, et al Rice RNA-dependent RNA polymerase 6 acts in small RNA biogenesis and spikelet development. Plant J. 2012;71(3):378–89. Epub 2012/03/27. 10.1111/j.1365-313X.2012.05001.x . 22443269

[35] Maniar JM, Fire AZ. EGO-1, a C. elegans RdRP, modulates gene expression via production of mRNA-templated short antisense RNAs. Curr Biol. 2011;21(6):449–59. Epub 2011/03/15. 10.1016/j.cub.2011.02.019 ; PubMed Central PMCID: PMCPmc3073447. 21396820 PMC3073447

[36] Zong J, Yao X, Yin J, Zhang D, Ma H. Evolution of the RNA-dependent RNA polymerase (RdRP) genes: duplications and possible losses before and after the divergence of major eukaryotic groups. Gene. 2009;447(1):29–39. Epub 2009/07/21. . 19616606 10.1016/j.gene.2009.07.004

[37] Sperling EA, Robinson JM, Pisani D, Peterson KJ. Where's the glass? Biomarkers, molecular clocks, and microRNAs suggest a 200-Myr missing Precambrian fossil record of siliceous sponge spicules. Geobiology. 2010;8(1):24–36. Epub 2009/11/26. 10.1111/j.1472-4669.2009.00225.x . 19929965

[38] Robinson JM, Sperling EA, Bergum B, Adamski M, Nichols SA, Adamska M, et al The identification of microRNAs in calcisponges: independent evolution of microRNAs in basal metazoans. Journal of experimental zoology Part B, Molecular and developmental evolution. 2013;320(2):84–93. Epub 2013/01/26. 10.1002/jez.b.22485 . 23349041

[39] Sempere LF, Cole CN, McPeek MA, Peterson KJ. The phylogenetic distribution of metazoan microRNAs: insights into evolutionary complexity and constraint. Journal of experimental zoology Part B, Molecular and developmental evolution. 2006;306(6):575–88. Epub 2006/07/14. 10.1002/jez.b.21118 . 16838302

[40] Peterson KJ, Dietrich MR, McPeek MA. MicroRNAs and metazoan macroevolution: insights into canalization, complexity, and the Cambrian explosion. Bioessays. 2009;31(7):736–47. Epub 2009/05/28. 10.1002/bies.200900033 . 19472371

[41] Prochnik SE, Rokhsar DS, Aboobaker AA. Evidence for a microRNA expansion in the bilaterian ancestor. Dev Genes Evol. 2007;217(1):73–7. Epub 2006/11/15. 10.1007/s00427-006-0116-1 . 17103184

