# Correction: miRNA Repertoires of Demosponges *Stylissa carteri* and *Xestospongia testudinaria*

**DOI:** 10.1371/journal.pone.0153731

**Published:** 2016-04-11

**Authors:** Yi Jin Liew, Taewoo Ryu, Manuel Aranda, Timothy Ravasi

The authors would like to correct the Author Contributions. The correct contributions are: Conceptualized the study: Y.J.L T.Ry M.A T.R. Contributed materials and processed the small RNA reads: T.Ry. Performed the miRNA predictions and produced figures: Y.J.L. Wrote the first draft of the manuscript: Y.J.L. M.A. Wrote the final version of the manuscript: Y.J.L T.Ry M.A T.R.
