# Supplementary figures and images for: miRNA Repertoires of Demosponges Stylissa carteri and Xestospongia testudinaria
Source: PLoS One. 2016 Feb 12;11(2):e0149080. doi: 10.1371/journal.pone.0149080 (PMC4752309; doi:10.1371/journal.pone.0149080)

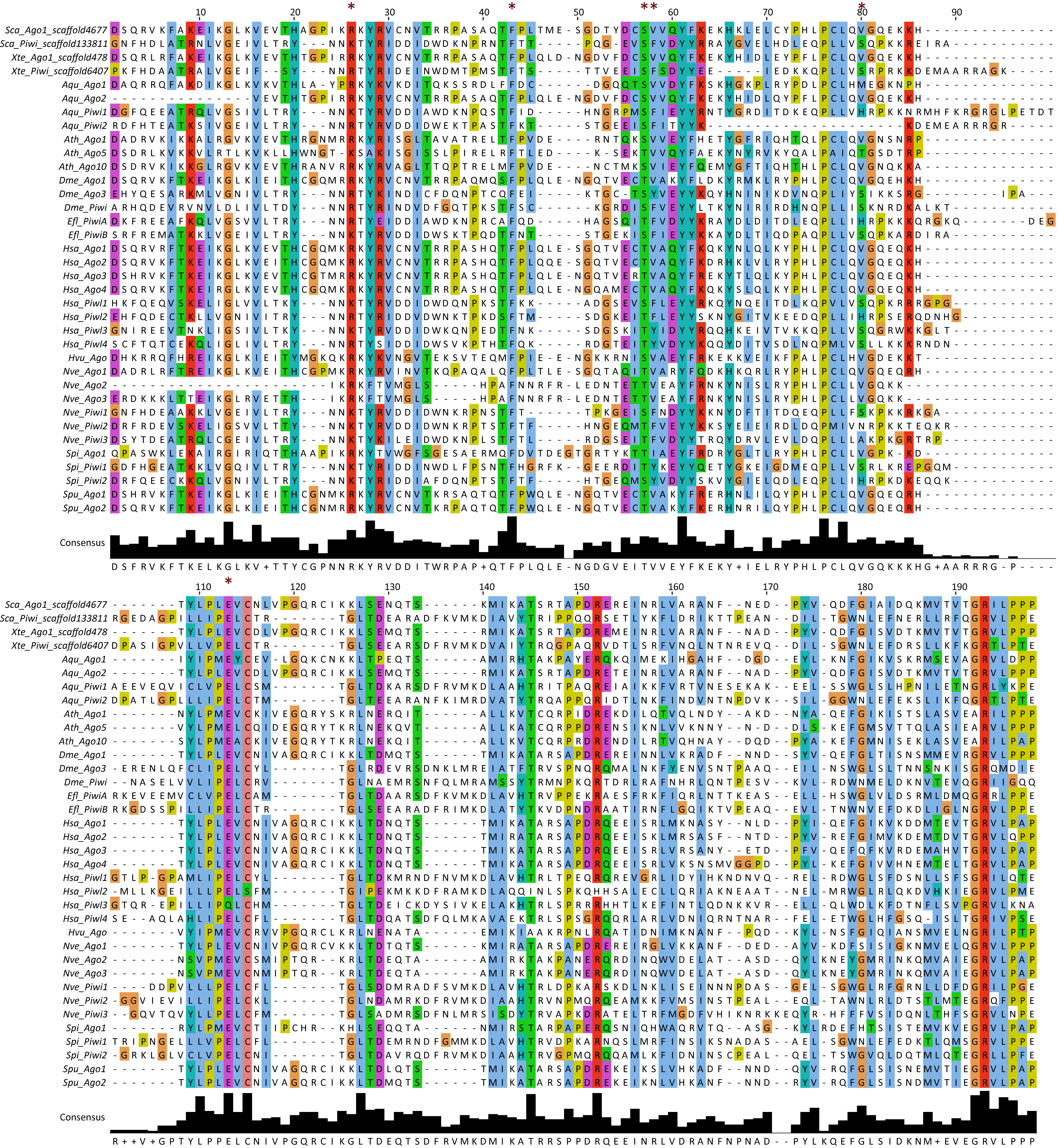

Supplement: S1 Fig — Of note are the strong conservation of phenylalanine (F) at position 43 and glutamate (E) at position 113. Mutations in the former affect RNA binding; mutations in the latter produce insoluble protein. Key residue positions are marked with red asterisks. (TIF) [file pone.0149080.s001.tif]

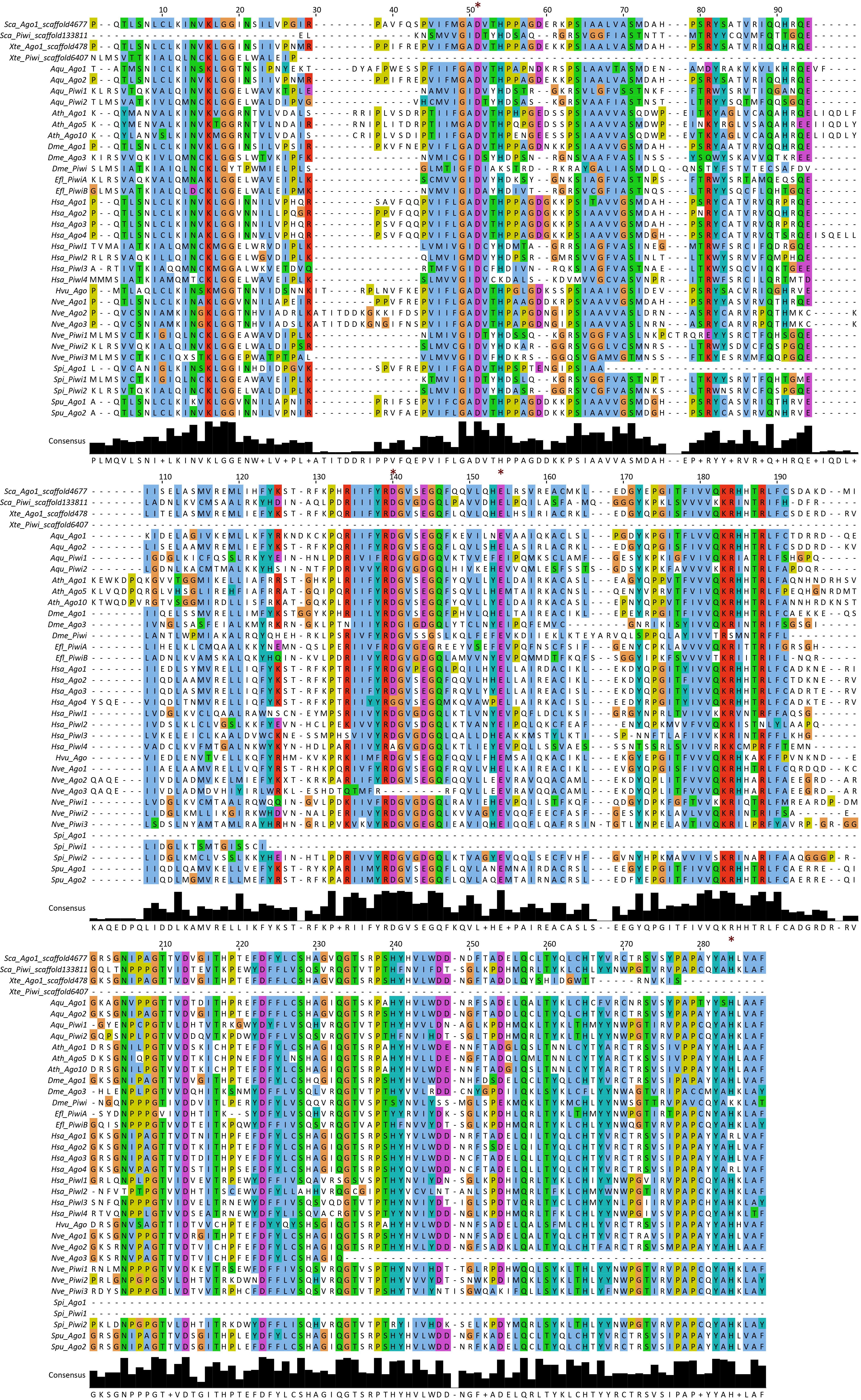

Supplement: S2 Fig — The catalytic DDX triad, which contributes to the slicing activity of the ribonuclease (marked in red asterisks), is located at positions 51, 140 and 284 or positions 51, 140 and 154. This triad is absent in the candidate Piwi homologue for X. testudinaria, likely due to it being truncated. (TIF) [file pone.0149080.s002.tif]

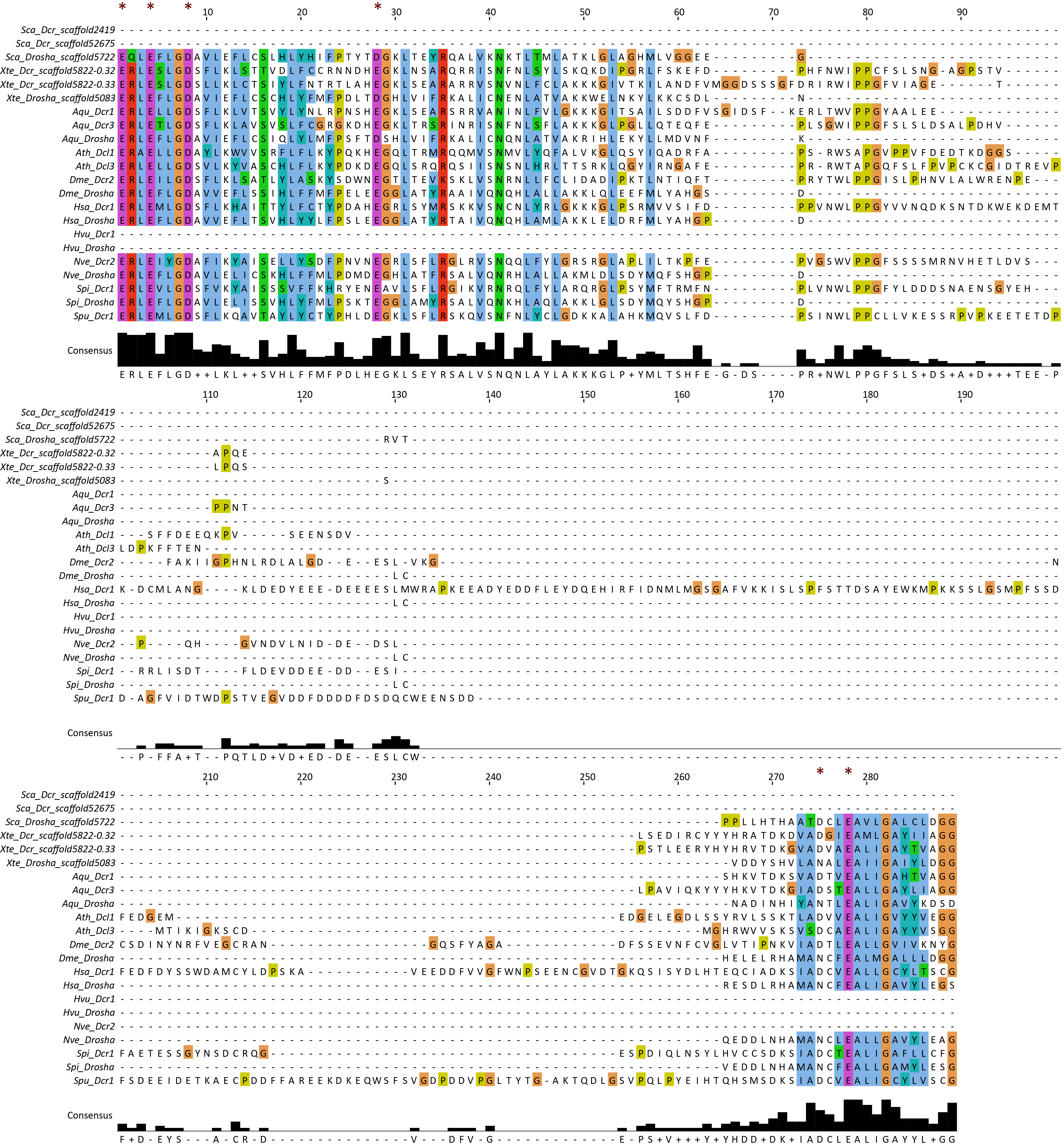

Supplement: S3 Fig — The acidic aspartate (D) and glutamate (E) residues are involved in the coordination of a divalent metal cation, and are well conserved in most of the candidate homologues. These residues are absent in the candidate Dicers for S. carteri, likely due to truncation. (TIF) [file pone.0149080.s003.tif]

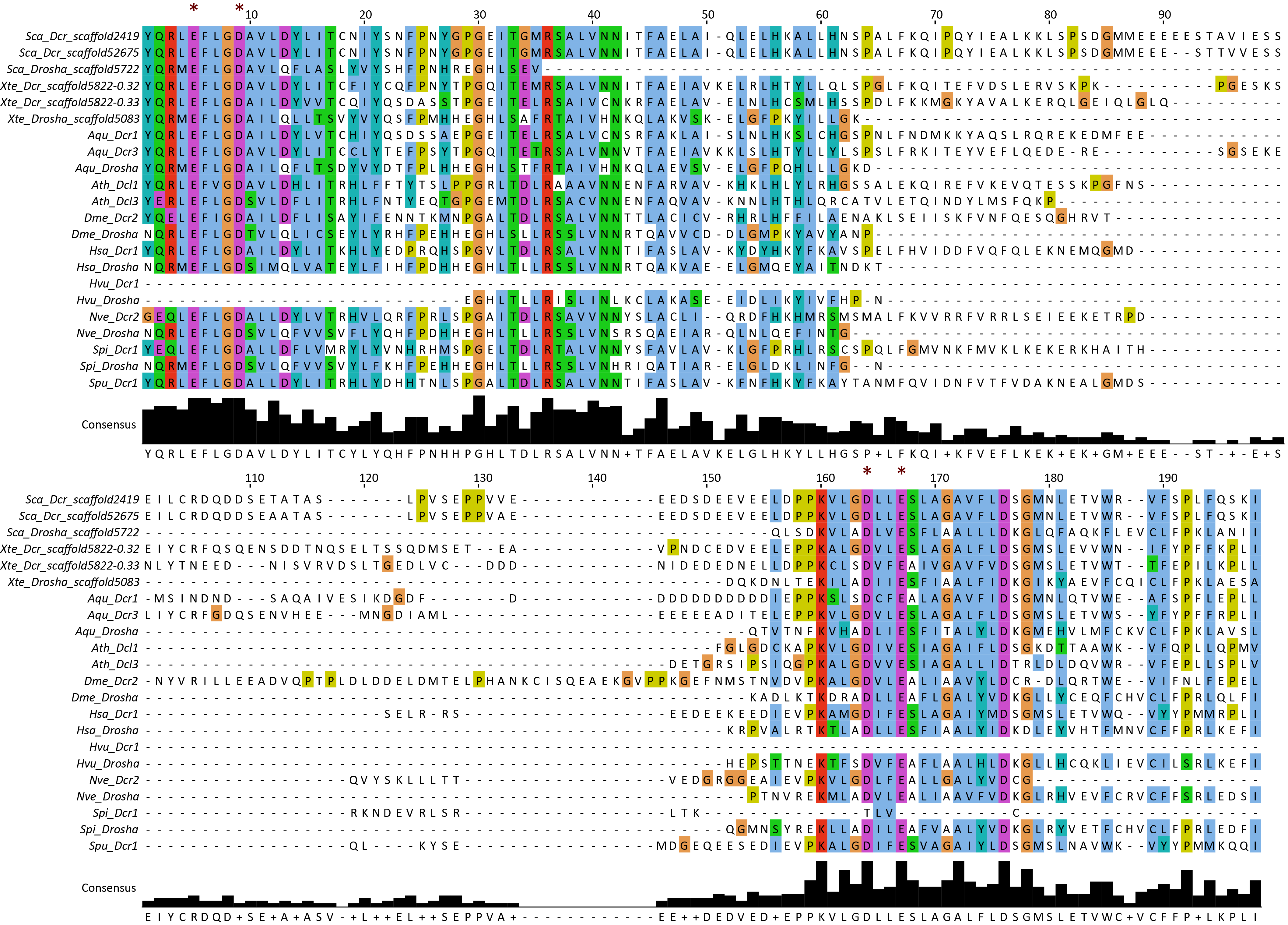

Supplement: S4 Fig — The aspartate (D) and glutamate (E) residues involved in the coordination of a divalent metal cation are perfectly conserved across all candidate Dicers and Drosha proteins. (TIF) [file pone.0149080.s004.tif]

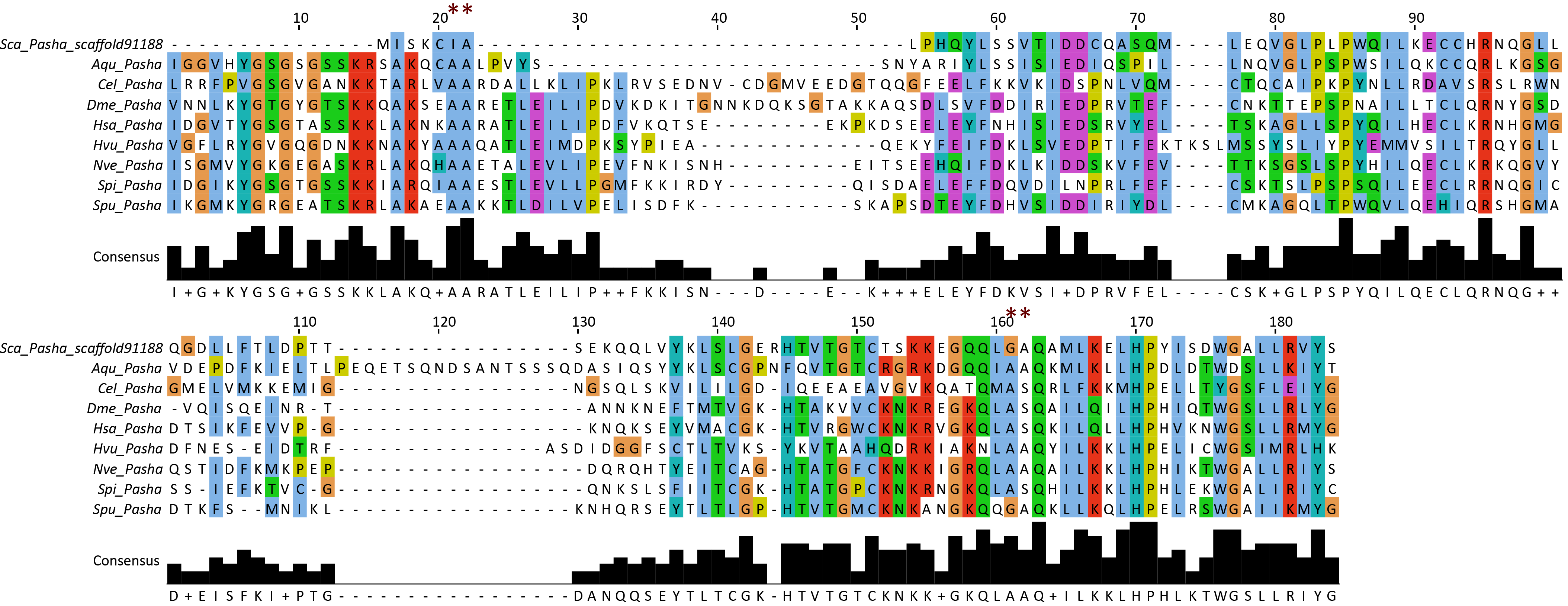

Supplement: S5 Fig — The conventional alanine/alanine pair (AA, positions 21 and 22) and alanine/serine pair (AS, positions 161 and 162) involved in the binding of dsRNA are not present in the candidate Pasha for S. carteri. Instead, for the former, S. carteri Pasha has an isoleucine/alanine pair that is not found in any other known Pasha; for the latter, it has a glycine/alanine pair, which is an exact match of S. purpuratus Pasha. (TIF) [file pone.0149080.s005.tif]

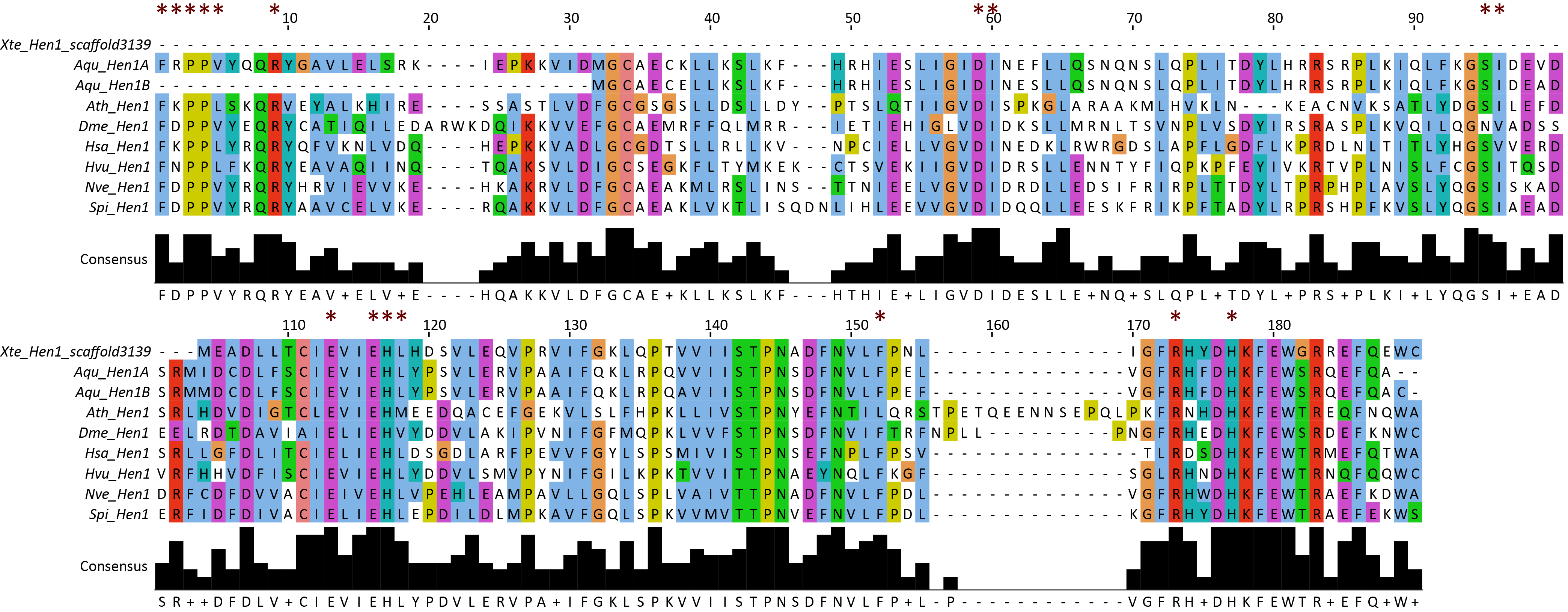

Supplement: S6 Fig — The residues involved in Mg2+ coordination (positions 113, 116, 117, and 118) are well-conserved across the aligned sequences; residues associated with the cofactor AdoHcy and 3' terminus (other positions marked by a red asterisk) are absent, likely due to the candidate being truncated. (TIF) [file pone.0149080.s006.tif]

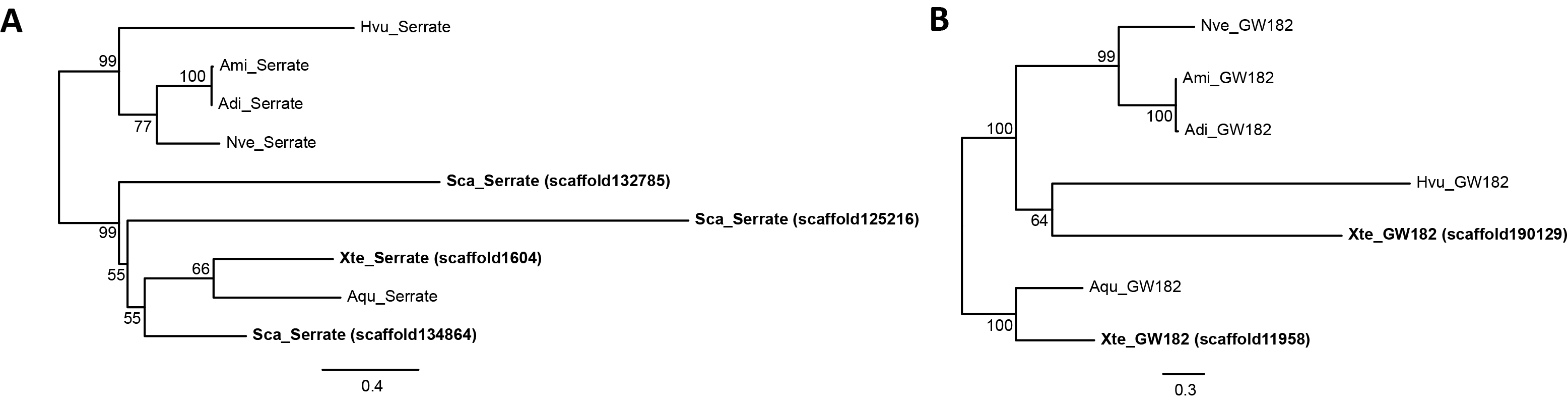

Supplement: S7 Fig — The (A) Serrate tree was built with the LG+G amino acid substitution model, while (B) GW182 was with the JTT+G model. (TIF) [file pone.0149080.s007.tif]

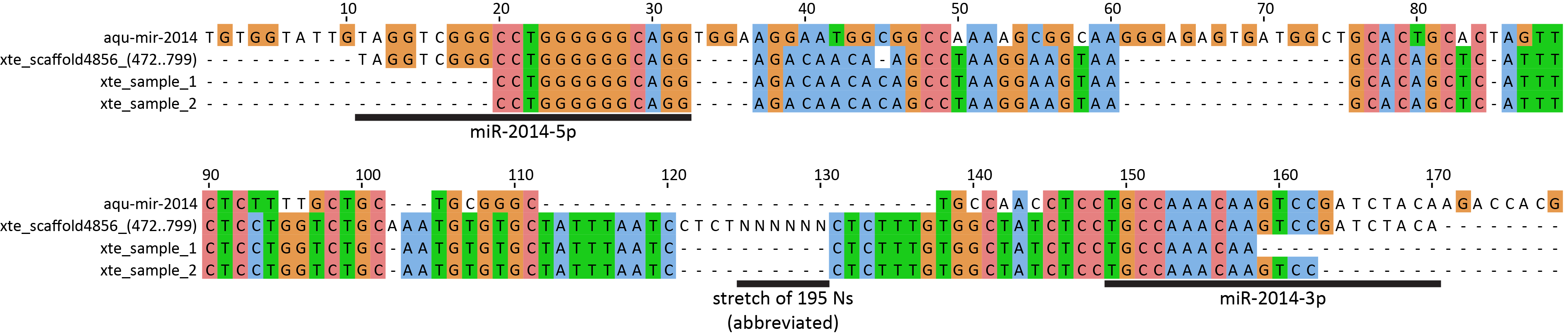

Supplement: S8 Fig — Primers corresponding to miR-2014-5p and the reverse complement of miR-2014-3p successfully amplified a ~100bp fragment in two X. testudinaria samples. Sanger sequences from the bands, when aligned against aqu-mir-2014 from A. queenslandica and the genomic sequence from X. testudinaria containing xte-mir-2014, indicate that the stretch of Ns in the X. testudinaria genome is an artefact from genome assembly, possibly hindering the in silico identification of this miRNA. (TIF) [file pone.0149080.s008.tif]
