# Supplementary material for: miRNA Repertoires of Demosponges Stylissa carteri and Xestospongia testudinaria
Source: PLoS One. 2016 Feb 12;11(2):e0149080. doi: 10.1371/journal.pone.0149080 (PMC4752309; doi:10.1371/journal.pone.0149080)
Supplement: S1 File — (DOCX) [file pone.0149080.s009.docx]

**S1 File:** **Candidate RNAi proteins in *S. carteri* and *X. testudinaria*.**

| ***S. carteri* (Sca)** | | | | | | | | | | |
| --- | --- | --- | --- | --- | --- | --- | --- | --- | --- | --- |
| **Protein annotation** | **Length** | **Key protein domains present (InterProScan)** | | | | | **Reverse BLAST to Swiss-Prot, top hit** | | |  |
|  |  | **Paz** | **Piwi** | **RNase III** | **dsRBD** | **MTase** | **Annotated function** | **Organism** | **e value** | **Inferred function** |
| scaffold2419 | 378 |  |  | 2 |  |  | Dicer-1 | Bos taurus | 1.10E-53 | Dicer |
| scaffold4677 | 630 | 1 | 1 |  |  |  | Argonaute-3 | Mus musculus | 0 | Argonaute |
| scaffold5722 | 356 |  |  | 2 |  |  | Drosha | Homo sapiens | 2.72E-86 | Drosha |
| scaffold52675 | 346 |  |  | 2 |  |  | Dicer-1 | Bos taurus | 5.14E-46 | Dicer |
| scaffold91188 | 174 |  |  |  | 1 |  | Pasha | Homo sapiens | 6.64E-20 | Pasha |
| scaffold133811 | 787 | 1 | 1 |  |  |  | Piwi-like-2 | Homo sapiens | 4.34E-151 | Piwi |
| ***X. tetsudinaria* (Xte)** | | | | | | | | | | |
| **Protein annotation** | **Length** | **Key protein domains present (InterProScan)** | | | | | **Reverse BLAST to Swiss-Prot, top hit** | | |  |
|  |  | **Paz** | **Piwi** | **RNase III** | **dsRBD** | **MTase** | **Annotated function** | **Organism** | **e value** | **Inferred function** |
| scaffold478 | 922 | 1 | 1 |  |  |  | Argonaute-2 | Rattus norvegicus | 0 | Argonaute |
| scaffold3139 | 294 |  |  |  |  | 1 | HEN1 | Xenopus tropicalis | 1.33E-43 | HEN1 |
| scaffold5083 | 775 |  |  | 2 |  |  | Drosha | Homo sapiens | 3.24E-124 | Drosha |
| scaffold5822-0.32 | 1587 |  |  | 2 |  |  | Dicer-1 | Bos taurus | 7.87E-143 | Dicer |
| scaffold5822-0.33 | 1754 |  |  | 2 |  |  | Dicer-like-2a | Oryza sativa | 2.97E-94 | Dicer |
| scaffold6407 | 639 | 1 | 1 |  |  |  | Piwi-like-1 | Gallus gallus | 5.23E-175 | Piwi |

# Protein sequences

## Argonautes

>Sca_Ago1_scaffold4677

LDFLCEVLELGNVDEQRRPLSDSQRVKFAKEIKGLKVEVTHAGPIKRKYRVCNVTRRPASAQTFPLTMESGDTYDCSVVQYFKEKHKLELCYPHLPCLQVGQEKKHTYLPLEVCNLVPGQRCIKKLSENQTSKMIKATSRTAPDREREINRLVARANFNEDPYVQDFGIAIDQKMVTVTGRILPPPLLQYGGKARVQAQPEKGVWDMRGKQFHKGVEIHSWTMVLFAAYRQCPEDKLRHFIYQLRRISSDAGMPITKDPFFLRYVSGVDTVEPIFRQLKQDRPELQLIMVVLPGKTPVYAEVKRVGDTLLGVATQCVQVRNVLRPSPQTLSNLCLKINVKLGGINSILVPGIRPAVFQSPVIFMGADVTHPPAGDERKPSIAALVASMDAHPSRYSATVRIQQHRQEIISELASMVREMLIHFYKSTRFKPHRIIFYRDGVSEGQFQQVLQHELRSVREACMKLEDGYEPGITFIVVQKRHHTRLFCSDAKDMIGRSGNIPAGTTVDVGITHPTEFDFYLCSHAGVQGTSRPSHYHVLWDDNDFTADELQCLTYQLCHTYVRCTRSVSYPAPAYYAHLVAFRARYHLQEREDKSSGDGSSASLQSEERSPAAMAQAVRVHEEINKVMYFA

>Xte_Ago1_scaffold478

MSGIGTPVDQVMKHWTPMDRFQPPPHHHHPPPTPVTLGLPPFPITRGTGGYLPTASTLPSPSITSSPSLTPSPGLTPTSMLPWTPSPIPMIHSTLNLQPPPRPNFGQVGRPIGLRANHFQVKIPTNTLYHYDVAIQPDKCPRRVNREIIEALIQTRNEYFEDQRPVFDGKKNLYSRKPLPGIGRERVEITVTLGGDGNRERAFKVSVKYVAQVNLALLESVLRGEISGAIPFEAIQALDVVMRHLPSMTYTPVGRSFFAPPEGEPYTLGNGREVWFGFHQSIRPSMWKMMMNIDVSATAFYKRQSVLNFLHEVLDIENDAIRRPLSDSQRLRFAKEIKGLKVEVTHTGPIRRKYRVCNVTRRPASAQTFPLQLDNGDVFDCSVVQYFKEKYHIDLEYPFLPCLQVGQEKKHTYLPLEVCDLVPGQRCIKKLSEMQTSRMIKATSRTAPDREMEINRLVARANFNADPYVQDFGISVDTKMVTVTGRVLPPPKLQYGGKARVQALPDRGVWDMRGKQFHYGVDVSIWAIVIFTTVKQCPEEKLRNFIFQLRKISQDAGMPFHHEPQYVRYIQDRLGVERVEAVEPLFRQLCIELEGLQLILVVLPGKTPVYAEVKRVGDTLLGVATQCVQTRNVNRTSPQTLSNLCLKINVKLGGINSIIVPNMRPPIFREPVIFMGADVTHPPAGDEKKPSIAALVASMDAHPSRYSATVRIQQHRQELITELASMVREMLIEFYKSTRFKPQRIIFYRDGVSEGQFLQVLQHELHSIRIACRKLEDGYQPGISFIVVQKRHHTRLFCSDERDRVGKSGNIPAGTTVDVGITHPTEFDFFLCSHAGVQGTSRPSHYHVLWDDNGFTADDLQYSHIDGWTTRNVKISSNINTIFYSSGDGSSASQQSEEPRSPAAMAQAVKVHEGMCRVMYFA

## Piwi

>Sca_Piwi_scaffold133811

MTGRAKGRARGRSRGGVQPEARRPGEPQPTPPSEQQYVGRGRGRAVTSAPPPAQQQQPPPKPPVQQMAEMGINGSAQQKPTAEQPPSRGPRGMPPPEPATRPEHITDKRGTSGTSIDVISNFVVLRNRPDCAIFQYNVSYSPQVDSRRRRVGMLAEQEALVGKXXXXVVLRNRPDCAIFQYNVSYSPQVDSRRRRVGMLAEQEALVGKVRAFDGMILYLPHRLPQDVTTVLTTTDNGQTQVTMTITLTNEVHANSPVCLQLFNILFRRILSKLEMKQIGRHYYSPKMPVKVPQHKLELWPGFITSILQYERNVMLCADISHKILRTDTVWDFLNELYTRARGNFHDLATRNLVGEIVLTRYNNKTYRIDDIDWDKNPRNTFTTPQGEVSFVDYYRRAYGVELHDLEQPLLVSQPKKREIRARGEDAGPILLIPELCTRTGLTDEARADFKVMKDIAVYTRIPPQQRSETLYKFLDRIKTTPEANDILTGWNLEFNERLLRFQGRVLPPETIYQKSTSVVIILPTNRKDRYDAIKKFCCVDHPGFKSHELKNSMVVGIDTYHDSAQRGRSVGGFIASTNTTMTRYYCQVMFQTTGQELADNLKVCMSAALRKYHDINAQLPDRIIIYRDGVGDGQLPAVVDHELPQILASFAMQGGGYKPKLSVVVVKKRINTRIFHDFRGQLTNPPPGTVIDTEVTKPEWYDFFLVSQSVRQGTVTPTHFNVIFDTSGLKPDHMQRLTYKLCHLYYNWPGTVRVPAPCQYAHKLAFLVGQSIHKEPHKALADRLYFL

>Xte_Piwi_scaffold6407

MASSLVAMSTRGGGRGRGVGRGRSRGGGALESRRPGEQPKGTPSEAQQQVGRGRGRAAAQIQQKPPSPTSEVTPQMQKMSLDGKGGGAVAGARTGPTKGDRPPRREIQISEPHTRPEHITDKRGAAGNEVDLVTNYVILKNRPGKAIYQYNVSYSPPVDSKSLRIALLRDHEESLIGKIRAFDGMILYLPHRLPNDTTEVVSKTKSDDTIKLTIKLTNELSANSPVCLQLFNVLFRRILAKLEMKQIGRHYYNPKASSTIPQHKLEVWPGFVTSILQYEQNVMLCADISHKILRTDTVLDVFFELQNEPKFHDAATRALVGEIFSYNNRTYRIDEINWDMTPMSTFTSTTVEEISFSDYYEEIEDKKQPLLVSRPRKKDEMAARRAGKDPASIGPVLLVPELCTRTGLSEEARSDFRVMKDVAIYTRQGPAQRVDTLSRFVNQLNTNREVQDILSGWNLEFDRSLLKFKGRTLPTETIFQKDAKFSYKPLEADWSRDMRGKHLINSISLREYNIIFSARDVGLAQDFIQSLKRVGPPMGIMVSDPRVNETRDNRTDSFLRTLSGSISPNTQLVIVILPNNRKDLYDAVKKLCCVDSPVPSQCIVARTLSKKQNLMSVTTKIALQLNCKLGGELWALEIP

## Dicer

>Sca_Dcr_scaffold2419

MGVKITKEGEPLSRKSTAKDASPKPPRAKRMKPSLPHASLTTEKAANTHLFFRNSSSILSEYFGPHPPTVLSVRQQLEVQRLLTVSSSSLHALHSHKIEKRIDWTFHDPSLLLQALTHASYTKNRVTDCYQRLEFLGDAVLDYLITCNIYSNFPNYGPGEITGMRSALVNNITFAELAIQLELHKALLHNSPALFKQIPQYIEALKKLSPSDGMMEEEEESTAVIESSEILCRDQDDSETATASLPVSEPPVVEEEDSDEEVEELDPPKVLGDLLESLAGAVFLDSGMNLETVWRVFSPLFQSKIDKFEGKIPVPPIQELFELEPNIQTKRSWKKGGRVRCMLTVVDRGIQVSAEASSHKAAKSAAARAALKKIKAQT

>Sca_Dcr_scaffold52675

MGVKITKEGEPLSRKSTAEDASPKPPRAKRMKPSVPHASLTAEKAANTHLFFRNSSSILSEYFGPHPPTVLSVRQQLEVQRLLTVSSSSLHALHSHKIEKRIDWTFHDPSLLLQALTHASYTKNRVTDCYQRLEFLGDAVLDYLITCNIYSNFPNYGPGEITGMRSALVNNITFAELAIQLELHKALLHNSPALFKQIPQYIEALKKLSPSDGMMEEESTTVVESSEILCRDQDDSEAATASLPVSEPPVAEEEDSDEEVEELDPPKVLGDLLESLAGAVFLDSGMNLETVWRVFSPLFQSKIDKFEGKIPVPPIQELFELEPNIQTKRYAVKKELYFLRIGCVCL

>Xte_Dcr_scaffold5822-0.32

MEEKSDTELTVARPYQLELLDAALDGDTIVLLGTGAGKTFIAVMLIKQLSYSIVSSYKEDSNAKRTVFLVNTVPLVTQQADYIRAHTPLKVGHYCGENGVDSWKQAKWNNELDQNNILVMTRQIFLDMLTHSYVNLSRINLIIFDECHHAVKNDAYVQIMKFYPKCPENNRPRILGLSASVISGKCKPQQLERKLKDLEETLHCRTETAGDLAEVAKYATNPDETLVCYSSRSLDSQTGILKPILDKIAEFLECQQKSKSKDSIDVKLRGYLEECIYALDNISITSAIEAVELMKIEIEDILDNHPNNWESKLTQMVLLNLDIFAVECKKALKNEGDDHSPKFTSLLHILADTNPCLSNSPDKVCSIVFVERRSTAVCLARLIQQHHDGCLGHLNCSFIVGHTGRGGRILEQGANMNVKKQQEVLRKFRTGQLNLLVATSVVEEGLDIPKCNLVVRYDFPKTFQSHVQSKGRARAKNSRYLLLVNREDYADRRSDLDCYFQLEKKLEEICHGREVPGEDEVEKRLKHLIEPYQPYGVDGPQMTIDGSLSLLHRYCQKLPHDRFTILAPVFEDENMGGGICQLDQIWYPILFAVKIQLPMNCPVKELRAGPPLPRVNLAKKAAALEAIKQLHIEGELDDHLKPISHKDVDSDEEDEVKMTEKKKTHAGTQRKVMYYRNEVAKCLHNSKPVPGDNHLYGFIMKLNGKLYPSQENSDQPYQTLGILTSKPIPYTKIPSFSLYIGRFEVSVSLVSLTNLSLASHEIQILQEFHSTLFKKIPLNIKFPTDVSNSDPIHREYLIVPVSILLDTNPLVYFLDNEIVQRVLAAGSGELQVPTWPCDWTNFIGTLVRKKYTEEFRHGCQYQLYEVTNIDDNETPLTNFPSDQSITYAQYFLHKYNYEIQDYTQPSLTCKPVSMGKNSLKLVSSRFKDQESEQERQKGSEIKLFPELCQIYPLSASFWKLCRCVPTIIYRIEALLVINELSDLISKETGIGCTSSYEYSTQPELSSAGQSDLAVRNLNSLFYWSTGEAVDPNTMPQLEQSAVMNGTLRKPDNALLLQSLTTKSANDSIDLERLESLGDSFLKLSTTVDLFCCRNNDHEGKLNSARQRRISNFNLSYLSKQKDIPGRLFSKEFDPHFNWIPPCFSLSNGAGPSTVAPQELSEDIRCYYYHRATDKDVADGIEAMLGAYIIAGGIEAGFKWLRWLGMKFSSPARPMSMDDDDQMDVEVRLRSASQESFMSIELSNDAAATIKNPLLIQNSSFVFHTQCQVPPPALLHPVDNADAIIKKMIFMCLPTSTSCNYTPLCEKINWTFKDPALLLQALTHPSFQKNHLTDSYQRLEFLGDAVLDYLITCFIYCQFPNYTPGQITEMRSALVNNITFAEIAVKELRLHTYLLQLSPGLFKQITEFVDSLERVSKPKPGESKSEIYCRFQSQENSDDTNQSELTSSQDMSETEAVPNDCEDVEELEPPKALGDVLESLAGALFLDSGMSLEVVWNIFYPFFKPLIDKFRDEGVPVHPIKKLLELDSSAIIEKRPSQTRSHRCSVKVVDKGIHVTCVAKTYKAAKVGAAKRAIEKIKELKTESNFNS

>Xte_Dcr_scaffold5822-0.33

LLTVMAEGVTSAAVVQGGGGVMEEEKKEVKQGRPYQVELVEYALRENTIVSLGTGAGKTFVAVMLIREMSHMTSEKFTEETGQRTIFLAHTVPLVKQQANYIRENTNLKVSDFCGDAKNVDSWSKETWYDHFNNNNVLVFSRAIFLDLLLKAFIKPRQLNLIIFDECHHATKNDPFVQIMKVIKDAPEEQRPRILGLSASLLGKKVKPGELEKGVRALETVLMSTARTTRDLSDMVKYATNPDEKICNFNSRVDEVAASLKCIIDPTLDFLRTKVDKRQVKSPIEETARNLLDDLQSILVDLGPASAAGFVSQAIRELKNTMKFSKPEDQWDNLLGCLVLSQLTIFETKSREFKHQYGKLQDSNKVHAMFLEIADMAVRSGEMDIAEDTSSELKSKKEVKTLRGIIFVERRHVASCLSKLIQEKSRSDPDLQHISCDYVVGHNVGQNSTILKKEARMKSKEQERVLCKFRRGKINLLVATSVIEEGVDVPKCNLVIRFDMPQNFRSYAQSKGRARDKPSTFLLLIGGDHQSDSLKDIENYHNLEKELIVLCQEDRKLPSEEEIQAKMADKIPPYMPYGPEGARETIFSPRYCQRLPCDEFTVLVPSFEVRKIPKDNPGNLGEVIYTEDEDEDPKFNYICSLSLPFNCPIKKIIESPPMGSKLQSHRAAAIFAIEELHKLKELDDIFKPTPLPDSDDEDVREEKKIALAGTDKRSQLYKNEVATVLSSCYPQEQSPDNFLYVFVIREKDSGILYQSQLEVESPKALGILLKKRLPHWKPPELTANFEHSQKDYIVVPVTINRMDGNEIKEVAIDYNIASAINDISEIQKFPDPPYSAEKFKNSLISVRHRDSISHSGLLELYRPMLDPSLSPLSPFPDTKFANFLEYYNNKYNYELKDLEQPGLICTRVSMSFLKVFTSRYTQDVLISQKNIPITLFPELVQIYPLSASFWRLLQCLPACLWRLESLALAEDFSGTVMLETGIGHLHYDTVLFTDTVVKGYVDAGFGDIPSKTFKVKGDVIPESINDIPSQLDNRGPDNGLILQALTPKGANDSIDLERLESLGDSLLKLCTSIYLFNTRTLAHEGKLSEARARRVSNVNLFCLAKKKGIVTKILANDFVMGGDSSSGFDRIRWIPPGFVIAGETLPQSPSTLEERYHYHRVTDKGVADVAEALIGAYTVAGGLQGGIAFMKWLGVKFDMPPDILKSTAEPFETENSKMSLLFVNSSAIFKSYFGEPKSPRFIPKNADDRNRMISQTARVQQRIHYQFKNHLLLIEALTHPSYARNNITDCYQRLEFLGDAILDYVVTCQIYQSDASSTPGEITELRSAIVCNKRFAELAVELNLHCSMLHSSPDLFKKMGKYAVALKERQLGEIQLGLQNLYTNEEDNISVRVDSLTGEDLVCDDDNIDEDEDNELLDPPKCLSDVFEAIVGAVFVDSGMSLETVWTTFEPILKPLLEKFKDNIPVPAIKELYEKIPTGLIVKREISSKAPLSGSGFHHGLNRDSPKRRLTHLGNLLKLNQHCIDTAFGFFKMALQLNLTRGRKSSIMDTACLYLVCRSEGTPHMLLDFSDVLQINVYSLGRAYLKLSTALHINPPAIDPCLYIHRFAHKLELGDKVHDVSMTALRLVARMKRDWIHHGRRPAGLCGASLLVSARLHNFNRSVREVARVVKLSEGTIRKRLGDFKDTPSSRLTIDEFLKIDLEQEHDPPCFLEAKKRVKQLYQTSSDMSGTSSQSQSQDDSLSQLLSEEDQSTE

## Drosha

>Sca_Drosha_scaffold5722

MRTTASNCSLRRPEPTTSSNAAPQKGLARLVSTVERLEAGEVADAENRIRNNEQLEFLGDAVLEFLCSLHLYHIFPTYTDGKLTEYRQALVKNKTLTMLATKLGLAGHMLVGGEEGRVTPPLLHTHAATDCLEAVLGALCLDGGLPEVQKMFARLVFPEEDLYDVWMNYSPDPLQQQYPDGDRHMIEQSESLQRLVTLEQSVGVQFTHIRLLARAFTHASVGYTNLTMGSYQRMEFLGDAVLQFLASLYVYSHFPNHREGHLSEVQLSDKVLADLVESFLAALLLDKGLQFAQKFLEVCLFPKLANIIKSSQLVDANTRLHQAVAHKCREEGSKFEMPVYRVLKVEGPSGKQQFTS

>Xte_Drosha_scaffold5083

MEVTPNMVLLSEVDINSLTPSTFYHQSQTPLSTRKATDQLCILHDHFKSTLLTPHESVHPTPVHSGLPYNSCISKTPLSNLSTRETESISYCYIMTIDPMINFELNGQTIVKDDCGQYYTFSGLLILSNTSLDYIPSVFFHQNNSNYCLSMKPHPIPMGFVIEDLLLFMKYLFINLLNLYDWFHEVLSLESHSIWIMPKFVRREGQVSYVASMATVLQSFMSSFVPFYLQRPSSLSEELQISRHYSGSLVYNEMKCPSALRIDRLNWKGQNEVNFVHFSTVSKTFDFSSAKRDYDNKEAKLKKLQREAVSEKLKRKDYFRLKQLRHELHSMRRSQKNKEEIIEVNCYGYKTTNVYSDLCQYALLCPIITNHIKFHFSLLTLEDNLQYHFSNKTLLELALTHPSYLSHHNGPPQILNVSSHCGIYRGNSQVAMTTYKSTPPKRPKSGIEGLCSAASECNWSDKESDRPRDNERLEFLGDAVIEFLCSCHLYFMFPDLTDGHLVIFRKALICNENLATVAKKWELNKYLKKCSDLNSVDDYSHVLANALEAIIGAIYLDGGLEESKKVVAKLLFPEEDLHKVWMACYRDPLQYENPNGDREMIVTTKLLQVFTSLEEAVGVKFTNIRLLARAFTHPSYGYTNLMLGDYQRMEFLGDAILQLLTSVYVYQSFPMHHEGHLSAFRTAIVHNKQLAKVSKELGFPKYILLGKDQKDNLTEKILADIIESFIAALFIDKGIKYAEVFCQICLFPKLAESAEGLQFLDAKTRLQHAIVYTWC

## Pasha

>Sca_Pasha_scaffold91188

MISKCIALPHQYLSSVTIDDCQASQMLEQVGLPLPWQILKECCHRNQGLLQGDLLFTLDPTTSEKQQLVYKLSLGERHTVTGTCTSKKEGQQLGAQAMLKELHPYISDWGALLRVYSESFARRKPLSRPLELPAAHHGSDSVKNVDQSVLKKLKQEMQKLADEAKDKEDTITKI

## HEN1

>Xte_Hen1_scaffold3139

MEADLLTCIEVIEHLHDSVLEQVPRVIFGKLQPTVVIISTPNADFNVLFPNLIGFRHYDHKFEWGRREFQEWCIKVCNCYNYLVQFDGVGDPPLEMSHLGHCSQIAIFTRQESDHNRDRDISLTDTPYEQIYECTFPHELNTPEQRLSNEAHYLLHVLFRRRTAQNEQKRHLLVSCTENGLESNDTGHTSIENNDENGRHQLDESLDSPFETSHSLIINLLELLDYPQIQRFTTNVDEIRQGHTSQIVLIYGLCAILYDELEDSETESVIEEASEDEEDIIDNIGYVESTEVWD
